# Differential Contribution of BLT_1_ and BLT_2_ to Leukotriene B_4_-Induced Human NK Cell Cytotoxicity and Migration

**DOI:** 10.1155/2015/389849

**Published:** 2015-12-01

**Authors:** Meng Wang, Nermine Mostafa El-Maghraby, Sylvie Turcotte, Marek Rola-Pleszczynski, Jana Stankova

**Affiliations:** Division of Immunology, Department of Paediatrics, Faculty of Medicine and Health Sciences, Université de Sherbrooke, Sherbrooke, QC, Canada J1H 5N4

## Abstract

Accumulating evidence indicates that leukotriene B_4_ (LTB_4_) via its receptors BLT_1_ and/or BLT_2_ (BLTRs) could have an important role in regulating infection, tumour progression, inflammation, and autoimmune diseases. In the present study, we showed that LTB_4_ not only augments cytotoxicity by NK cells but also induces their migration. We found that approximately 30% of fresh NK cells express BLT_1_, 36% express BLT_2_, and 15% coexpress both receptors. The use of selective BLTR antagonists indicated that BLT_1_ was involved in both LTB_4_-induced migration and cytotoxicity, whereas BLT_2_ was involved exclusively in NK cell migration, but only in response to higher concentrations of LTB_4_. BLT_1_ and BLT_2_ expression increased after activation of NK cells with IL-2 and IL-15. These changes of BLTR expression by cytokines were reflected in enhanced NK cell responses to LTB_4_. Our findings suggest that BLT_1_ and BLT_2_ play differential roles in LTB_4_-induced modulation of NK cell activity.

## 1. Introduction

Human natural killer (NK) cells with the CD3^−^ CD56^+^ phenotype comprise 10–15% of peripheral blood lymphocytes. They constitute a major component of the innate immune system especially in response to transformed and infected cells [1–3]. Even though priming is not necessary for NK cells to perform their cytolytic function, proinflammatory cytokines, such as IL-2 [4, 5] and IL-15 [6], can induce NK cell proliferation, cytotoxicity, or cytokine production. Chemokine-induced NK cell migration may explain the redistribution of NK cells from the bone marrow and lymph nodes to blood and other organs [7]. In addition to chemokines, NK cells respond to other chemoattractants such as N-formyl-methionyl-leucyl-phenylalanine (f-MLP), casein, and C5a [8].

Leukotriene B_4_ (LTB_4_) is a potent lipid mediator of allergic and inflammatory reactions, in addition to modulating immune responses [9, 10]. LTB_4_ is a major chemoattractant of granulocytes [11, 12] and can be responsible for T cell recruitment in asthma [13–15]. Two human LTB_4_ cell-surface receptors, BLTRs, high-affinity BLT_1_ and low-affinity BLT_2_, were cloned and identified in 1997 and 2000, respectively [16, 17]. It has been demonstrated that BLT_1_ expression is high in peripheral blood leukocytes and lower in other tissues, whereas BLT_2_ expression is ubiquitous in most human tissues with lower expression in peripheral blood leukocytes [18]. Studies using BLT_1_^−/−^ mice and specific BLT_1_ antagonists have demonstrated that BLT_1_ plays critical roles in both host defence and many inflammatory diseases by mediating multiple activities of LTB_4_, including inflammatory cell recruitment [19, 20], prolongation of inflammatory cell survival [21, 22], and activation of inflammatory cell functions [23, 24]. Recent studies with BLT_2_^−/−^ mice showed that BLT_2_ is involved in autoantibody-induced severe inflammatory arthritis [25] but is protective in DSS-induced colitis by enhancing epithelial cell barrier functions [26]. However, the functions and biological activity of BLT_2_ in lymphocytes are not completely known at this time.

It has been shown that LTB_4_ could augment the cytolytic function of human NK cells [27–29] and induce T lymphocyte recruitment to inflammatory sites [13–15]. These observations led us to examine whether LTB_4_ was chemotactic for NK cells and to define the contribution of BLT_1_ and/or BLT_2_ to NK cell migration and cytolysis in response to LTB_4_. We first determined BLT_1_ and BLT_2_ expression in NK cells, at both the mRNA and protein levels, and then studied the differential contribution of these receptors in LTB_4_-induced NK cell migration and cytotoxicity. We also evaluated the modulation of BLT_1_ and BLT_2_ expression after cytokine stimulation and the subsequent effect on NK cell responses to LTB_4_.

## 2. Materials and Methods

### 2.1. Antibodies and Reagents

Mouse anti-human CD56 and CD3 antibodies and 7AAD were purchased from BD Biosciences (Mississauga, ON, Canada). FITC-conjugated goat anti-rabbit IgG (GAR-FITC) and DTAF-conjugated streptavidin (SA-FITC) were from Jackson ImmunoResearch Laboratories (West Grove, PA, USA). Polyclonal rabbit anti-human BLT1R and BLT2R antibodies, LTB_4_, CAY10583, U75302, and LY255283 were from Cayman Chemical (Ann Arbor, MI, USA). Isotype control rabbit IgG was from InterSciences (Markham, ON, Canada). Biotinylated mouse anti-human BLTR antibody and isotype control were from AbD SeroTec (Raleigh, NC, USA). Human IL-2 and IL-15 were purchased from PeproTech (Dollard des Ormeaux, QC, Canada). MIP-1*α* was from Abcam (Cambridge, MA, USA). All other chemical agents were obtained from Sigma-Aldrich (Oakville, ON, Canada) unless otherwise mentioned.

### 2.2. Cell Culture

Peripheral blood mononuclear cells (PBMCs) and lymphocytes (PBLs) were isolated as described previously [30]. Briefly PBMCs were isolated from healthy volunteers' peripheral blood using density gradient centrifugation with Ficoll-Paque PLUS (GE healthcare) and PBLs were collected after monocyte depletion of PBMCs by adherence. Human NK cells were purified from fresh PBLs using Macs magnetic system (Miltenyi Biotec, Cambridge, MA, USA) with human NK cell enrichment kits (StemSep, Vancouver, BC, Canada), according to the manufacturer's directions. Enrichment routinely resulted in greater than 95% purity as determined by cytometric analysis with anti-CD56 antibodies. PBLs or NK cells (2 × 10^6^ cells/mL) were cultured in RPMI 1640 (Invitrogen, Burlington, ON, Canada) with 80 IU/mL penicillin G (Novopharm, Toronto, ON, Canada), and 100 *μ*g/mL streptomycin and 5% FBS (PAA, Etobicoke, ON, Canada) in the absence or presence of IL-2 or IL-15, 10 ng/mL, in a humidified atmosphere with 5% carbon dioxide at 37°C. The antagonists, U75302 10 *μ*M and LY255283 50 *μ*M, were added 30 minutes prior to stimulation with LTB_4_.

### 2.3. Semiquantitative End Point or Real-Time PCR Analysis

After appropriate treatment, total cellular RNA was isolated using TRIzol reagent (Invitrogen). After treatment with RNasin (Promega, Madison, WI, USA) and DNase kit (Fermentas, Burlington, ON, Canada) to exclude genomic DNA contamination, 1 *μ*g of RNA was converted to cDNA with oligo(dT) (Fermentas) and M-MLV reverse transcriptase (Promega) in a volume of 20 *μ*L.

*End point RT-PCR* was performed in a final volume of 50 *μ*L containing 2 *μ*L cDNA, 1 *μ*M primer, and the reaction buffer of Taq DNA polymerase kit (Feldan, Quebec, QC, Canada), using a Biometra thermocycler (Montreal Biotech, Montreal, QC, Canada) using an initial denaturation step at 95°C for 2 min, 24 cycles (for GAPDH) or 32 cycles (for BLT_1_/BLT_2_) of 30 s denaturation at 95°C/30 s annealing at 58°C/30 s extension at 72°C, and a final 8 min extension at 72°C. Positive controls of cloned human BLT_1_ or BLT_2_ cDNA were described previously [31]. Negative controls, in which the reverse transcription step was omitted, confirmed that the PCR products reflected mRNA levels rather than contaminating genomic DNA. PCR products (10 *μ*L) were electrophoresed in a 1.2% (w/v) agarose gel and visualized with ethidium bromide. Densitometric quantification was done with NIH ImageJ software.

*Real-time PCR* was performed with Rotor-Gene 3000 system (Corbett Research, Concorde, NSW, Australia) using the SYBR Green I detection method. Each sample for the real-time PCR consisted of 1 *μ*L of cDNA, 1 *μ*M primer, 2.5 mM MgCl_2_, the reaction buffer of Taq DNA polymerase kit (Feldan), and 0.8 *μ*L of SYBR Green I (1/1000 stock dilution; Molecular Probes, Invitrogen) in a reaction volume of 25 *μ*L. The cycling program consisted of an initial denaturation at 95°C for 5 minutes, 45 cycles of amplification conditions as follows: 95°C (30 s), 58°C (30 s), and 72°C (30 s) and a final extension at 72°C for 6 min. Comparison of the expression of each gene between its control and stimulated states was determined with the delta-delta (ΔΔ)Ct, according to the following formula:

(1) ΔΔCt=Ct  G.O.I.Ctl−Ct  H.K.G.Ctl−Ct  G.O.I.STIM.−Ct  H.K.G.STIM..

Results were then transformed into fold variation measurements: fold increase = 2^ΔΔCt^. Each experiment was performed in duplicate.

PCR primers (IDT, Coralville, IA, USA) were designed with Primer3, and their sequences are as follows: GAPDH (housekeeping gene, 246 bps) 5′-GAT GAC ATC AAG AAG GTG GTG AA-3′ (forward), 5′-GTC TTA CTC CTT GGA GGC CAT GT-3′ (reverse); hBLT_1_ (216 bps) 5′-GTT TTG GAC TGG CTG GTT GC-3′ (forward), 5′-GGT ACG CGA GGA CGG GTG TG-3′ (reverse); hBLT_2_ (183 bps) 5′-GAG ACT CTG ACC GCT TTC GT-3′ (forward), 5′-AAG GTT GAC TGA GTG GTA GG-3′ (reverse).

### 2.4. Flow Cytometry

#### 2.4.1. Cell-Surface Staining

Freshly isolated cells (1 × 10^6^) were suspended in 5 *μ*L PBS-2% BSA and labelled with 5 *μ*L anti-BLTR-Biotin or isotype antibodies for 30 minutes on ice. After washing with PBS, cells were incubated with SA-FITC, anti-CD3-APC, and anti-CD56-PE antibodies for 30 minutes, then washed, and resuspended in 200 *μ*L PBS.

#### 2.4.2. Intracellular Staining

Freshly isolated cells (1 × 10^6^) were fixed with 2% paraformaldehyde and permeabilized with 0.1% saponin at room temperature. Cells were then incubated for 15 minutes with human IgG to block binding to Fc receptors, resuspended in 100 *μ*L PBS-2% BSA, and labelled with polyclonal anti-BLT_1_ Ab (1 : 2000 dilution), polyclonal anti-BLT_2_ Ab (1 : 1000 dilution), or isotype control for 30 minutes at room temperature. After washing with PBS, cells were incubated with GAR-FITC, anti-CD3-APC, and anti-CD56-PE antibodies for 20 minutes at room temperature, then washed, and resuspended in 200 *μ*L PBS.

#### 2.4.3. Flow Cytometry Analysis

100,000 events/sample were recorded and analyzed with FACSCalibur (BD Biosciences) and Flowjo Software (Treestar, Ashland, OR, USA). Each experiment was performed in duplicate.

### 2.5. Chemokinesis and Chemotaxis Assays

NK cell chemotactic activity was evaluated using a modified Boyden chamber assay. A volume of 200 *μ*L RPMI 1640-2% BSA with MIP-1*α* (1 ng/mL) and graded concentrations of LTB_4_ or control (medium or EtOH) alone was added to the lower chamber. A volume of cells (6 × 10^5^), which were prestained with anti-CD3-FITC/CD56-PE antibodies and preincubated with or without antagonists, in 200 *μ*L RPMI 1640-2% BSA only or LTB_4_ (for the chemokinesis assay), was added to the upper chamber. The two chambers were separated by a 5 *μ*m pore size polycarbonate filter (Neuroprobe, Gaithersburg, MD). After a 3-hour incubation, migrating cells were collected from the lower chamber and on the lower side of the filter for counting by flow cytometry with fixed time acquisition. Each experiment was performed in triplicate. The number of migrating NK cells was quantitated by counting CD3^−^ CD56^+^ cells in the migrating population. The results were then converted to a migration index (MI): MI = mean number of cells migrating to chemoattractant/mean number of cells migrating to control (EtOH or medium).

### 2.6. Cytotoxicity Assay

Target cells, K562 (an erythroleukemia cell line, ATCC), were labelled with 0.1 *μ*M CFSE for 5 minutes at room temperature, washed twice with PBS-2% FBS, and suspended at 5 × 10^5^ cells/mL in RPMI 1640-5% FBS. Effector cells, PBLs or enriched NK cells, were preincubated with or without antagonists. Effector and target cells were then coincubated at indicated effector : target ratios in a final volume of 200 *μ*L. Graded concentrations of LTB_4_ or vehicle control were used during the 2-hour cytotoxicity assay. Target cells alone were incubated in medium to measure spontaneous cell death. After a 2 h incubation, 2 *μ*L 7AAD was added to every sample and kept on ice for 15 min. Samples were immediately acquired by flow cytometry (BD FACSCalibur). The analysis was performed on gated cells that fell within the CFSE positive population (K562). Within this population of cells, we quantified the 7AAD labeling for each sample. Cytotoxicity was determined as

(2) cytotoxicity%=CFSE+7AAD+CFSE+×100%−spontaneous  death.

Each experiment was performed at least in duplicate.

### 2.7. Statistical Analysis

Data are presented as mean ± SEM. Statistical tests (Student's *t*-test, one-way ANOVA, or 2-way ANOVA, as appropriate) were performed using GraphPad Prism 5.0 (GraphPad Prism Software, San Diego, CA). *P* < 0.05 was considered significant.

## 3. Results

### 3.1. BLT_1_ and BLT_2_ Expression on NK Cells

**Figure 1:**
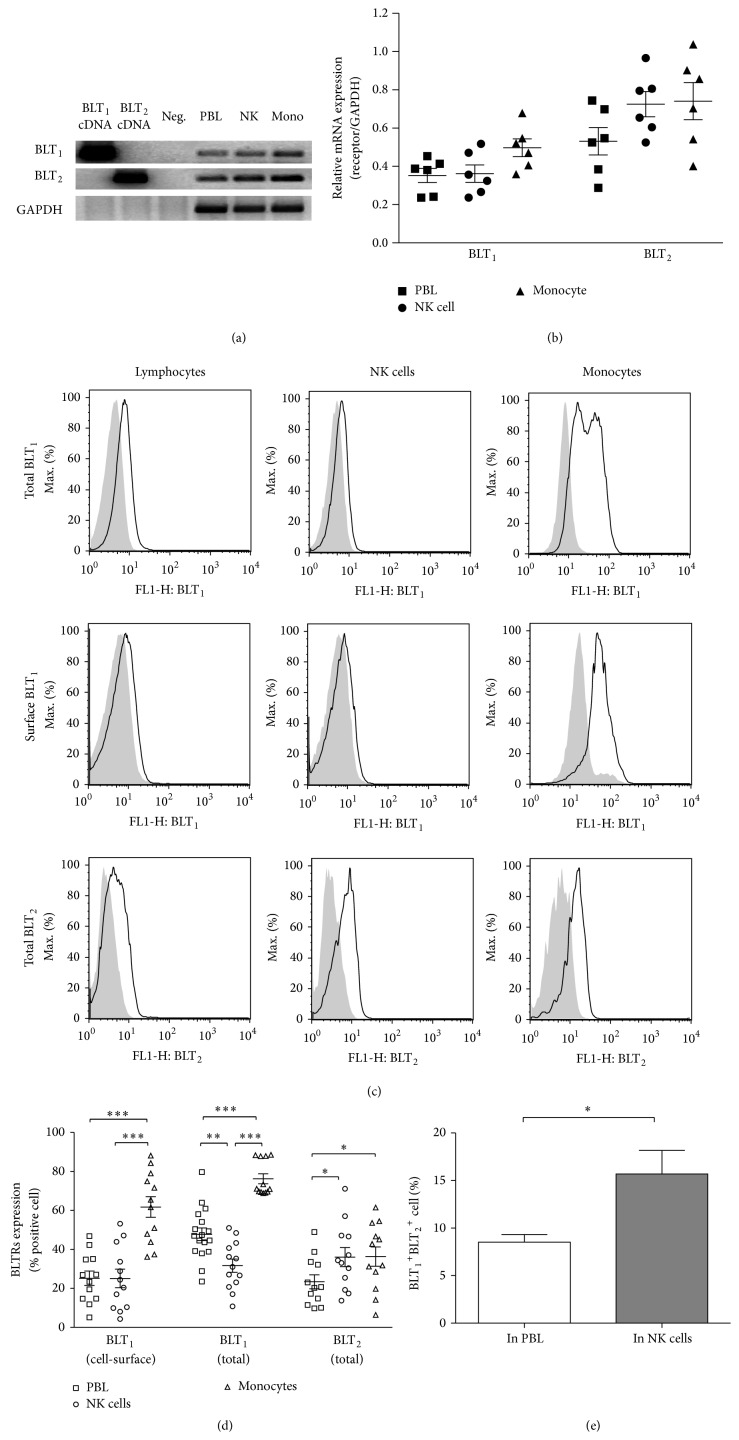
Expression of BLT_1_ and BLT_2_ in NK cells. RT-PCR of BLT_1_, BLT_2_, and GAPDH from PBLs, isolated NK cells, and monocytes were analyzed by agarose gel electrophoresis (a):* lanes 1* and* 2*, human BLT_1_ and BLT_2_ cDNAs as positive controls;* lane 3*, polymerase-only, RT-negative samples for each oligonucleotide pair;* lane 4*, PBL mRNA;* lane 5*, NK cell mRNA; and* lane 6*, monocyte mRNA. (b) Expression levels of BLT_1_ and BLT_2_ mRNA were determined by densitometry and the ratio to GAPDH (the mean ± the SEM) from six donors is shown. (c) Whole cell and cell-surface expression of BLT_1_ (top and middle rows, resp.) and whole cell expression of BLT_2_ (bottom row) on PBLs, NK cells, and monocytes were measured by flow cytometry as described in Section 2. In the overlaid histogram graphs, grey background indicates control histograms, where cells were incubated with Ig isotype, and solid line indicates cells incubated with anti-BLTR antibodies. (d) Results represent the percentage of BLTR positive cells as population comparison analysis. Bars represent means ± SEM of ten to seventeen independent experiments. (e) Coexpression of BLT_1_ and BLT_2_ was measured as the percentage of events that were BLT_1_ and BLT_2_ double positive, gating on PBLs and NK cells (CD56^+^), respectively. Bar graphs represent means ± SEM of four independent experiments. ^*∗*^ *P* < 0.05; ^*∗∗*^ *P* < 0.01; ^*∗∗∗*^ *P* < 0.001, paired Student's *t*-test.

Initially it was believed that BLT_1_ was expressed only in phagocytes (granulocytes, eosinophils, and macrophages) [17, 32–34]. However, BLT_1_ mRNA was found in differentiated CD4^+^  T_H_ cells and CD8^+^  T_EFF_ cells and the LTB_4_-BLT_1_ pathway was found to be involved in inflammation-induced T_H_ and T_EFF_ cell recruitment [35, 36]. We, and others, have reported that LTB_4_ could augment NK cell cytotoxicity [27, 37–39]. Thus, we sought to determine the pattern of BLTR expression in NK cells. We assessed BLT_1_ and BLT_2_ mRNA expression by RT-PCR in each population of cells, PBLs, enriched NK cells, and monocytes (Figure 1(a)). Densitometric analysis of six donors' data, shown in Figure 1(b), indicated that BLT_1_ and BLT_2_ mRNA expression was similar in PBLs, NK cells, and monocytes, with a tendency for higher BLT_2_ mRNA expression in these cell populations.

BLTR expression on fresh PBMCs was then evaluated by flow cytometry. Polyclonal anti-BLT_1_ and anti-BLT_2_ antibodies, which are directed toward an intracellular (C-terminus) domain of the receptor, were used to evaluate total (intracellular and extracellular) expression in permeabilized cells. In addition, a monoclonal anti-BLT_1_ antibody, which recognizes the N-terminal receptor epitope (extracellular), was used to evaluate cell-surface expression. Histogram graphs in Figure 1(c) illustrate the BLTR expression from a representative donor on lymphocytes, CD56^+^ NK cells, and monocytes, respectively. Figure 1(d) illustrates a compilation of individual donors and indicates that BLT_1_ receptors are expressed both intracellularly and on the cell-surface of all three cell populations. Around 25.19% ± 3.69% PBLs expressed BLT_1_ on the cell-surface, whereas almost 50% PBLs expressed BLT_1_ when intracellular receptors were taken into account (47.76% ± 3.23%). The discrepancy was smaller in NK cells and monocytes, as the expression of BLT_1_ on cell-surface (25.09% ± 4.75% and 61.76% ± 5.27%, resp.) was around 80% of total expression (31.64% ± 3.39% and 76.23% ± 2.56%). Although the antibodies used for cytometry were different, it appeared that the expression of BLT_2_ protein was lower than that of BLT_1_ in PBLs and monocytes, but it was similar in NK cells. Due to a lack of an antibody directed at the extracellular region of BLT2, we could not evaluate differences between its cell-surface and its total cellular expression. Interestingly, more NK cells (15.69% ± 2.49%) expressed both BLTRs than PBLs (8.53% ± 0.79%) (Figure 1(e)). Our findings of the heterogeneous expression of BLTR in NK cells led us to study the involvement of BLT_1_ and/or BLT_2_ in LTB_4_-mediated effects in these cells.

### 3.2. NK Cell Migration Response to LTB_4_

**Figure 2:**
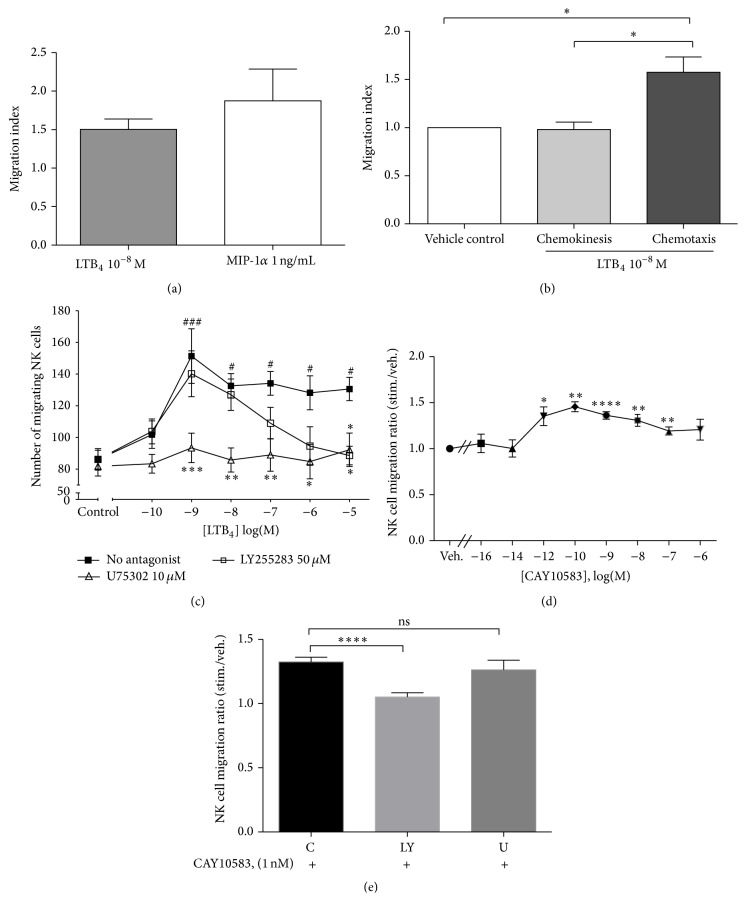
NK cell migration in response to LTB_4_. (a) Bar graphs represent NK cell migration in response to MIP*α*, 1 ng/mL, and LTB_4_, 10^−8^ M. (MI: migration index, number of cells migrating in response to stimulus divided by number of cells migrating in response to control medium. Graph represents mean ± SEM of four independent experiments.) (b) Results illustrate the comparison of chemokinesis and chemotaxis to 10^−8^ M LTB_4_. Spontaneous migration in the presence of vehicle alone (ethanol 0.0033%) was normalized to 1. Bar graphs represent means ± SEM of four independent experiments, ^*∗*^ *P* < 0.05 paired Student's *t*-test. (c) PBLs were preincubated without antagonist (■), with U75302 10 *μ*M (∆) or LY255283 50 *μ*M (□) for 30 minutes at 37°C, before a chemotaxis assay with graded concentrations of LTB_4_ or vehicle control. The number of migrating cells was measured by FACS, gating on the NK cell population (CD3^−^ CD56^+^). Each curve represents mean ± SEM of five independent experiments. ^#^ *P* < 0.05, and ^###^ *P* < 0.001 by one-way ANOVA with Dunnett posttest to vehicle control. ^*∗*^ *P* < 0.05, ^*∗∗*^ *P* < 0.01, and ^*∗∗∗*^ *P* < 0.001 by two-way ANOVA with Bonferroni posttests to no-antagonist data. (d) NK cell migration in response to graded concentrations of CAY10583. Data are expressed as means ± SEM of ratios of migrating cells in response to CAY10583 versus vehicle (*n* = 8), ^*∗*^ *P* < 0.05, ^*∗∗*^ *P* < 0.01, ^*∗∗∗*^ *P* < 0.001, and ^*∗∗∗∗*^ *P* < 0.0001. (e) NK cell migration in response to 10^−9 ^M CAY10583 in the absence or presence of LY255283 (LY) or U75302 (U). ^*∗∗∗∗*^ *P* < 0.0001, *n* = 5.

LTB_4_ is a potent neutrophil chemoattractant and recruits neutrophils to inflammatory sites in skin or lung by directing cell migration [40, 41]. Recent studies demonstrated that LTB_4_ can also induce migration of mast cells [42], dendritic cells [43], and T cells [35, 36, 44]. Thus, we investigated whether LTB_4_ could induce migration of human NK cells. As shown in Figure 2(a), LTB_4_ induced a 1.5-fold migration of NK cells at 10^−8 ^M, and the magnitude of migration was similar to that induced by MIP-1*α*, a potent chemoattractant of NK cells [7]. This increase in chemotaxis was due to directional migration as LTB_4_ did not modulate NK cell chemokinesis (Figure 2(b)). NK cells migrated in response to a wide range of LTB_4_ concentrations, from 10^−9^ to 10^−5 ^M, with a maximum at 10^−9 ^M LTB_4_ (Figure 2(c)). In order to study the contribution of the two receptors to chemotaxis, we used selective antagonists. We found that LTB_4_-induced chemotaxis was abolished following preincubation with the BLT_1_ antagonist, U75302 at 10 *μ*M, for 30 minutes. However, the BLT_2_ antagonist, LY255283, blocked LTB_4_-induced chemotaxis only at the highest concentrations of the ligand. Moreover, the selective BLT_2_ agonist CAY10583 was capable of inducing significant NK cell migration (Figure 2(d)) and this migration was only blocked by LY255283 (Figure 2(e)).

Our data suggest that the high-affinity BLT_1_ receptor mediates most of the LTB_4_-induced chemotactic activity in NK cells, with the lower affinity BLT_2_ receptor participating preferentially when LTB_4_ concentrations are very high.

### 3.3. BLT_1_ but Not BLT_2_ Mediates LTB_4_-Induced NK Cell Cytotoxicity

**Figure 3:**
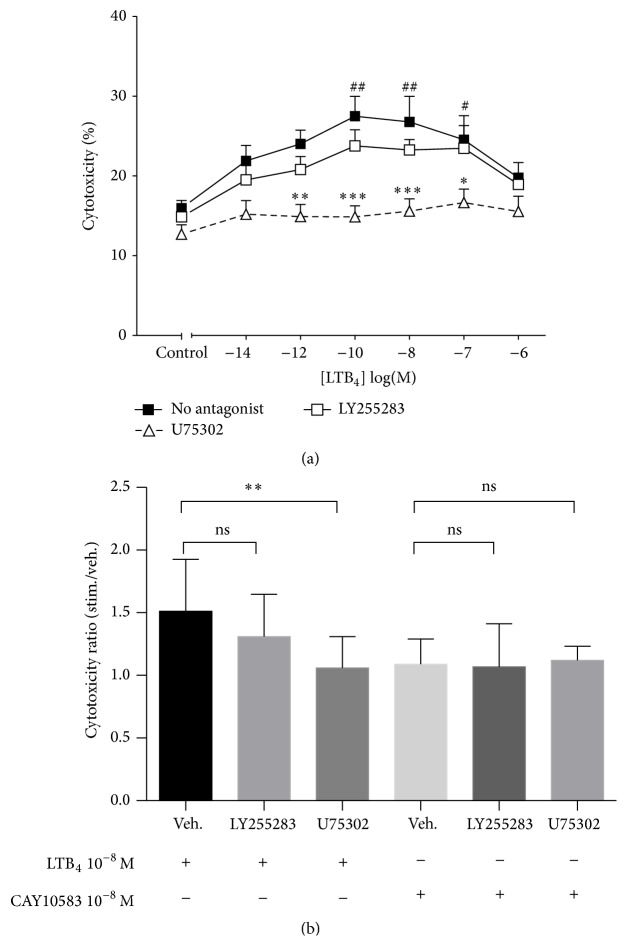
(a) LTB_4_ enhances NK cell cytolytic function via the BLT_1_ receptor. PBLs (effector cells) were preincubated without antagonist (■) or with U75302 10 *μ*M (∆) or LY255283 50 *μ*M (□) for 30 minutes at 37°C and then were coincubated with CFSC-labelled K562 cells at* E* :* T* = 50 : 1 ratio in the presence of graded concentrations of LTB_4_. Cytotoxicity was measured using the percentage of 7AAD positive events gating on K562 cells. Each curve represents mean ± SEM of seven independent experiments. ^#^ *P* < 0.05, and ^##^ *P* < 0.01 by one-way ANOVA with Dunnett posttest to controls. ^*∗*^ *P* < 0.05, ^*∗∗*^ *P* < 0.01, and ^*∗∗∗*^ *P* < 0.001 by two-way ANOVA with Bonferroni posttests to no-antagonist data. (b) Effects of LTB_4_ or CAY10583 on NK cell cytotoxicity in the absence or presence of LY255283 or U75302. Data are expressed as means ± SEM of cytotoxicity ratios. ^*∗∗*^ *P* < 0.01 using Student's paired *t*-test; *n* = 9.

To determine which of the two receptors was necessary for the LTB_4_-induced effect on NK cell cytotoxic function, we again used the selective antagonists. LTB_4_ significantly enhanced NK cell cytolytic activity at 10^−10 ^M to 10^−7 ^M, with a maximal effect between 10^−10 ^M and 10^−8 ^M of LTB_4_ (Figure 3(a)). Preincubation with U75302 at 10 *μ*M for 30 minutes abrogated the enhanced cytotoxicity. In contrast, LY255283 did not significantly block LTB_4_-induced cytotoxicity. Moreover, the selective BLT_2_ agonist Cay10583 failed to affect the level of NK cell activity (Figure 3(b)), suggesting that BLT_2_ was not involved in LTB_4_-induced cytotoxicity.

### 3.4. Modulation of LTB_4_ Receptor Expression by IL-2 and IL-15

**Figure 4:**
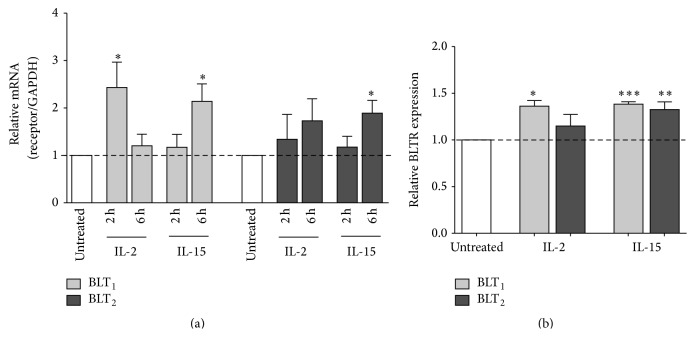
Modulation of BLTR expression on human NK cells by IL-2 and IL-15. (a) NK cells were incubated for 2 or 6 h in the absence or presence of IL-2 (50 ng/mL) or IL-15 (10 ng/mL). Total RNA was then extracted and analyzed by q-PCR. Bar graphs represent mean ± SEM of five independent experiments. ^*∗*^ *P* < 0.05, by one-way ANOVA with Dunnett posttest to untreated cells (control). (b) PBLs were incubated for 18 h in the absence or presence of IL-2 or IL-15. Total expression of BLT_1_ (light grey) and BLT_2_ (dark grey) on NK cells was measured by FACS and expressed as fold induction relative to untreated cells. Bar graphs represent mean ± SEM of seven independent experiments. ^*∗*^ *P* < 0.05, ^*∗∗*^ *P* < 0.01, and ^*∗∗∗*^ *P* < 0.001 by paired Student's *t*-test, treated versus untreated cells.

Pro- or anti-inflammatory cytokines can modulate BLT_1_ expression in human monocytes, with decreased expression induced by IFN-*γ* and TNF*α*, and increased expression stimulated by IL-10 and dexamethasone [45]. TNF*α* and dexamethasone also regulate BLT_1_ expression in neutrophils [21, 46]. Therefore, we sought to investigate whether IL-2 and IL-15, two NK-activating cytokines, could regulate BLTR expression. Real-time quantitative PCR was used to examine BLT_1_ and BLT_2_ mRNA expression in purified NK cells, incubated with IL-2 or IL-15 for 2 or 6 hours. IL-2 was found to induce a 2.4-fold increase of BLT_1_ expression after a 2 h stimulation but did not significantly increase BLT_2_ expression. IL-15 induced a 2.1-fold increase of BLT_1_ and a 1.9-fold increase of BLT_2_ expression after a 6 h incubation (Figure 4(a)). BLTR protein expression was then examined in NK cells incubated with IL-2 or IL-15 for 18 h. IL-2 only increased expression of BLT_1_, whereas IL-15-activated NK cells showed approximately 1.4- and 1.3-fold higher expression of both BLT_1_ and BLT_2_, respectively (Figure 4(b)).

### 3.5. Regulation of LTB_4_-Induced NK Cell Migration by IL-2 and IL-15

**Figure 5:**
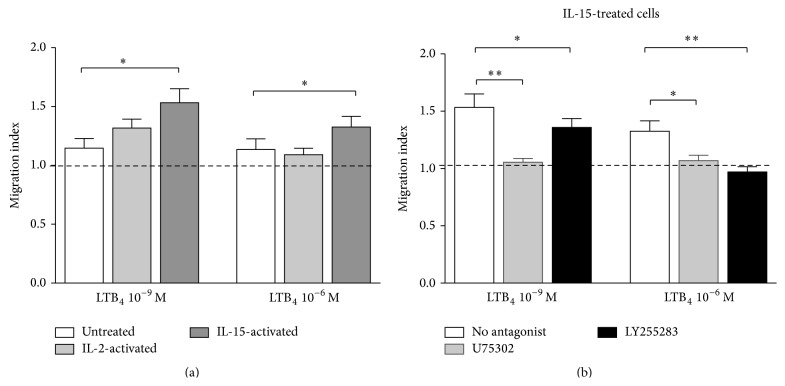
Effect of IL-2 or IL-15 pretreatment on LTB_4_-induced NK cell migration. (a) PBLs were incubated for 18 h in the absence or presence of IL-2 (50 ng/mL) or IL-15 (10 ng/mL). Cells were stained with anti-CD3 and anti-CD56 antibodies and then placed in a chemotaxis assay in response to LTB_4_ 10^−9 ^M or 10^−6 ^M. Migrating CD3^−^ CD56^+^ cells were counted by FACS, and results were expressed as migration index (LTB_4_/vehicle). Bar graphs represent mean ± SEM of seven independent experiments. ^*∗*^ *P* < 0.05 by paired Student's *t*-test, treated versus untreated cells. (b) IL-15-activated PBLs were preincubated with or without U75302 or LY255283 before chemotaxis assays. Migrating CD3^−^ CD56^+^ cells were counted by FACS, and results were expressed as migration index (LTB_4_/vehicle). Bar graphs represent mean ± SEM of seven independent experiments. ^*∗*^ *P* < 0.05 by paired Student's *t*-test.

Dexamethasone-treated human neutrophils show a higher response to LTB_4_ in chemotaxis, as a result of enhanced BLT_1_ expression [21]. For this reason, we hypothesized that IL-2 and IL-15 may increase the chemotactic response of NK cells to LTB_4_. We treated PBLs with IL-2, IL-15, or medium for 18 h and NK cell migration in response to 10^−9 ^M and 10^−6 ^M LTB_4_ was examined (Figure 5(a)). IL-15 induced a significant augmentation of NK cell migration to both concentrations of LTB_4_. However, IL-2 did not significantly increase NK cell chemotactic response to LTB_4_ at either concentration. As illustrated in Figure 5(b), U75302 abolished the migration of IL-15-activated NK cells in response to both concentrations of LTB_4_; meanwhile, LY255283 totally abrogated the migration of IL-15-activated NK cells to higher concentration LTB_4_ (10^−6^ M) but only partially blocked the migration to lower concentration (10^−9^ M). Our data suggest that IL-15-enhanced chemotactic response of NK cells to LTB_4_ is also predominantly dependent on BLT_1_ signaling.

### 3.6. Higher Response of IL-2- and IL-15-Activated NK Cells to LTB_4_ in Cytotoxicity

**Figure 6:**
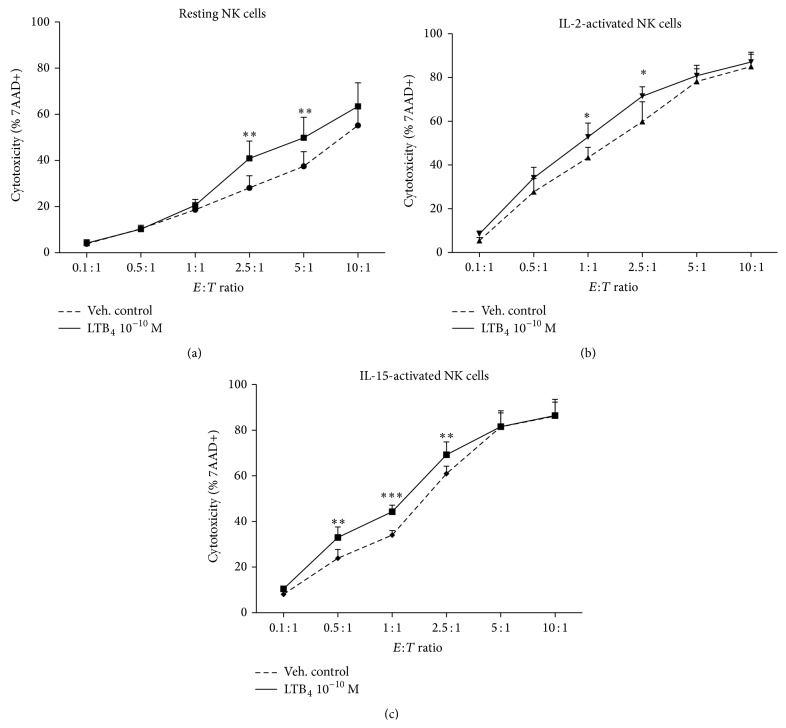
Effect of IL-2 and IL-15 pretreatment on LTB_4_-induced NK cell cytotoxicity. Resting NK cells (a), IL-2-activated NK cells (b), and IL-15-activated NK cells (c) were coincubated with CFSE-labelled K562 cells at different* E* :* T* ratios for a 2 h cytotoxicity assay in the presence of LTB_4_ 100 pM (solid lines) or vehicle control (dotted lines). The killing ability was measured using the percentage of 7AAD positive K562 cells. Each curve represents the means ± the SEM of three independent experiments. ^*∗*^ *P* < 0.05, ^*∗∗*^ *P* < 0.01, and ^*∗∗∗*^ *P* < 0.001 by two-way ANOVA with Bonferroni posttests to vehicle control.

We next tested whether IL-2- and IL-15-activated NK cells could also increase their cytotoxic responses to LTB_4_. We treated purified NK cells with IL-2, IL-15, or medium for 18 h and then coincubated NK cells and K562 target cells at a graded effector : target cell ratio from 0.1 : 1 to 10 : 1. As shown in Figure 6, LTB_4_ induced a significant augmentation of cytotoxicity in resting NK cells at* E* :* T* ratios of 2.5 to 5 : 1. IL-2- and IL-15-activated NK cells showed an enhanced cytotoxicity in response to LTB_4_ at lower* E* :* T* ratios of 1 : 1 and 2.5 : 1 and 0.5 : 1 to 1 : 1, respectively. The BLT_1_ antagonist U75302, but not the BLT_2_ antagonist LY255283, blocked NK cell responsiveness to LTB_4_ (data not shown), suggesting that IL-2- and IL-15-induced upregulation of BLT_1_ expression may be involved in the augmented cytotoxic response to LTB_4_ by NK cells.

## 4. Discussion

In the present study we investigated the differential involvement of BLT_1_ and BLT_2_ receptors in LTB_4_-induced chemotaxis and cytotoxic activity of NK cells.

Previously, we and others have shown that LTB_4_ increases NK cell cytotoxicity in several species [37, 39, 47]. However, BLTR expression in NK cells was still unclear. We found that the BLT_1_ receptor was expressed on human NK cells, which is in contrast to the findings of Pettersson et al. [48] who reported that no CD56^+^ cells were BLT_1_-positive, although they found low expression on some CD16^+^ cells. Interestingly, we found that the expression of BLT_1_ was quite variable from individual to individual. The highest BLT_1_ cell-surface expression on PBL and NK cells was 10 times higher than the lowest one. Moreover, we found expression of both mRNA and protein, which confirmed BLT_1_ and BLT_2_ expression in human NK cells. Meanwhile, we found that the expression of both receptors in monocytes and lymphocytes was similar to the findings of Islam et al. [13] and Yokomizo et al. [33]. The fact that protein expression of BLTR was different, especially that of BLT_1_, between PBLs, NK cells, and monocytes whereas mRNA expression was similar, suggests that there might be not only transcriptional regulation of BLT_1_ expression [49], but also posttranscriptional regulation of expression of these proteins. The higher expression of BLT_2_ mRNA in comparison to BLT_1_ mRNA might be due to the special gene structure, as the open reading frame of BLT_2_ is found in the promoter of BLT_1_ [49].

NK cells are rapidly distributed throughout the body in order to exercise their effector functions. However, the chemotactic signals responsible for their migration are not well known. We show here that LTB_4_ could induce a similar level of NK cell migration as MIP-1*α*, which has been shown as one of the most potent NK cell chemoattractants [7]. This suggests that LTB_4_ may induce early and rapid NK cell recruitment and trafficking to inflammatory sites, given that LTB_4_ is synthesized very rapidly [10] compared to chemokines. The peak of NK cell migration was in response to a low (1 nM) concentration of LTB_4_, which was lower than the optimal concentration inducing CD8^+^ T cell migration (10 nM) [36] or mast cell migration (100 nM) [42].

Similar to T cells [13–15] and neutrophils [11, 12], the BLT_1_ receptor was the principal signal transducer of LTB_4_-induced chemotaxis of human NK cells. However, unlike in the other cell types, BLT_2_ plays a partial role in the chemotactic response of NK cells to LTB_4_. This agrees with the observations of Yokomizo et al., who found that both BLT1 and BLT_2_ could mediate LTB_4_-induced cell migration in BLT1-BLT2 cotransfected CHO cells [33]. Since 12-HHT, a fatty acid derived from the COX pathway, may be the more effective agonist for BLT_2_ [50], it may also potentially be involved in NK cell chemotaxis.

On the other hand, our results show that BLT_2_ is probably not involved in LTB_4_-induced augmentation of NK cell cytotoxicity since the BLT_1_, but not the BLT_2_, antagonist could block all the effects of LTB_4_ and since the BLT_2_ agonist CAY10583 had no effect on NK cell cytotoxicity.

BLT1 and BLT2 belong to the seven transmembrane domains, G-protein-coupled receptor (GPCR) family. In some GPCRs, dimerization is associated with better receptor activation [51], and it has been shown that the BLT_1_:BLT_1_ homodimer does show higher affinity for LTB_4_ than the monomer [52]. However, BLT_2_ monomers have been shown to be more efficient at activating G proteins than the dimers [53]. In the present study, both BLT_1_ and BLT_2_ antagonists could block NK cell migration to higher concentrations of LTB_4_. This might suggest that, in NK cells, which coexpress the two receptors, BLT1:BLT2 heterodimers might exist, and although heterodimers of BLT receptors have not been investigated, it would not be surprising if one of the monomers influenced signal transduction [54].

We also observed that higher concentration of LTB_4_ seemed to induce a lower NK cell migration and cytotoxicity. It has been shown that rapid agonist-induced internalization and desensitization are important characteristics of BLT1 activation [55]. Thus, possibly, desensitization and internalization could reduce receptor responses at higher concentrations of LTB_4_.

It is now well established that pro- and anti-inflammatory cytokines as well as microbial products can modulate the expression of BLT1 in monocytes [45] and neutrophils [21]. In the present study, we found that cytokines which increase NK cell activation and cytotoxicity could also modulate BLT expression. We found that IL-15 upregulated both BLT_1_ and BLT_2_ expression, but IL-2 only increased BLT_1_ protein expression. These cytokines have also been shown to upregulate chemokine receptors in human NK cells [56, 57]. Although IL-2 and IL-15 belong to the same *γ*c family of cytokines [6], IL-15R *α*-chain has a much broader tissue distribution than the IL2R *α*-chain, which is absent in NK cells [58]. IL-15 efficiently engages IL-2/15R*βγ* with IL-15R*α* for signal transduction, leading to the rapid upregulation of receptors for C-C chemokines, whereas IL-2 is unable to do so [57], in parallel with unaffected BLT_2_ expression.

Moreover, IL-2 and IL-15 augmented NK cell responses to LTB_4_ in terms of chemotaxis and cytolytic function. Preincubation with IL-2 did not increase NK cell migration to the same degree as treatment with IL-15, especially at higher concentration of LTB_4_, suggesting that BLT_2_ may be involved. The lower effect of IL-2 on BLT expression may be the consequence of the lack of IL-2R*α*, which results in a receptor with lower affinity (IL-2R*βγ*) for IL-2, and this in turn could result in a weaker modulation of BLT_2_ expression. However, IL-2- and IL-15-activated NK cells showed a similar augmentation in LTB_4_-induced cytotoxicity, which was mediated by BLT_1_, possibly due to the equivalent upregulation of BLT_1_ expression by both cytokines.

We previously showed that LTB_4_ augments IL-2R*β* expression in CD56^+^ NK cells and induces their responsiveness to 100-fold lower concentrations of IL-2 in terms of cytolytic activity [30]. Moreover, IL-15R*α* mRNA expression was augmented in NK cells after a 30-minute stimulation with 100 nM LTB_4_ (data not shown). The evidence that cytokines and leukotrienes can increase each other's receptor expression suggests that IL-2, IL-15, and LTB_4_ may synergize to augment NK cell migration to tumour or infection sites and enhance cytotoxic responses.

In conclusion, functional BLT_1_ and BLT_2_ receptors are expressed in human NK cells and mediate, to a different degree, LTB_4_-induced chemotaxis and cytotoxicity. IL-2- and IL-15-dependent modulation of BLTR expression can upregulate the response of NK cells to LTB_4_.

## Abbreviations

7AAD: 7-Aminoactinomycin D
ADCC: Antibody-dependent cellular cytotoxicity
APC: Allophycocyanin
BLTR: LTB_4_ receptor
CFSE: Carboxyfluorescein succinimidyl ester
FITC: Fluorescein isothiocyanate
GAPDH: Glyceraldehyde-3-phosphate dehydrogenase
LTB_4_: Leukotriene B_4_
NK cell: Natural killer cell
PBLs: Peripheral blood lymphocytes
PE: R-Phycoerythrin.
